# Air-pollution prediction in smart city, deep learning approach

**DOI:** 10.1186/s40537-021-00548-1

**Published:** 2021-12-22

**Authors:** Abdellatif Bekkar, Badr Hssina, Samira Douzi, Khadija Douzi

**Affiliations:** 1 FSTM, University Hassan II, Casablanca, Morocco; 2 FMPR, University Mohammed V, Rabat, Morocco

**Keywords:** Air-pollution, *PM*_2.5_, Deep learning, Forecasting, LSTM, GRU, CNN-LSTM

## Abstract

Over the past few decades, due to human activities, industrialization, and urbanization, air pollution has become a life-threatening factor in many countries around the world. Among air pollutants, Particulate Matter with a diameter of less than $2.5 \mu m$ ($PM_{2.5}$) is a serious health problem. It causes various illnesses such as respiratory tract and cardiovascular diseases. Hence, it is necessary to accurately predict the $PM_{2.5}$ concentrations in order to prevent the citizens from the dangerous impact of air pollution beforehand. The variation of $PM_{2.5}$ depends on a variety of factors, such as meteorology and the concentration of other pollutants in urban areas. In this paper, we implemented a deep learning solution to predict the hourly forecast of $PM_{2.5}$ concentration in Beijing, China, based on CNN-LSTM, with a spatial-temporal feature by combining historical data of pollutants, meteorological data, and $PM_{2.5}$ concentration in the adjacent stations. We examined the difference in performances among Deep learning algorithms such as LSTM, Bi-LSTM, GRU, Bi-GRU, CNN, and a hybrid CNN-LSTM model. Experimental results indicate that our method “hybrid CNN-LSTM multivariate” enables more accurate predictions than all the listed traditional models and performs better in predictive performance.

## Introduction

The increase in the percentage of the urban population in the world shows that people more and more are moving to cities. According to United Nations (UN), the urban population as of 2020 is about 56.15% [1]. And it is expected that it will become 68% of the world’s population will live in urban cities by 2050 [2]. The growth of urbanization and industrialization causes several problems logistics, health care, and air quality. In order to resolve these issues, and improve the quality of its citizens’ lives, The smart city concept was created by integrating Information and Communication Technology (ICT), and fixed/mobile sensors. These last are installed within the city to observe real human practice. This concept become an endless source of urban data.

In the last decades, the frequent occurrence of smog caused by the increase in industrialization has harshly brought environmental pollution to its serious peak. That is, it becomes more severe than ever before. One of the hazardous pollutants is a fine particulate matter whose size is $2.5\,\upmu \text {m}$ or less, also known as $PM_{2.5}$. Such particle results in serious health damage. According to the World Health Organization WHO, almost 90% of people breathe polluted air that exceeds the limits of WHO guidelines in terms of air quality [3], bringing about respiratory problems [4, 5], moreover, even a few hours to weeks of short-term exposure to $PM_{2.5}$ can trigger cardiovascular disease-related mortality and events [6]. The Global Burden of Diseases GBD identified that Exposure to $PM_{2.5}$ contributed to 4.2 million deaths and 115.1 million disability-adjusted life years (DALYs) globally in 2015 [7] with an increase in 2017 (4.58 million deaths and 142.52 million DALYs) [8]. This poor air quality not only threatens the health and lives of individuals but the economies as well. The report carried out by the Organization for Economic Cooperation and Development OECD has shown that air pollution could cost 1% of world Gross domestic product GDP [9].

Due to the coronavirus pandemic (COVID-19), the epidemic center in China is the first to announce a lockdown on January 23, 2020. after, other countries did the same to reduce the spread of the Severe Acute Respiratory Syndrome Coronavirus 2 (SARS-CoV-2). Universally the COVID-19 lockdown creates a unique and precious opportunity to evaluate and to understand human activities, and the factors affecting air pollutants. Many studies have reported the environmental effects of lockdown policies on $PM_{2.5}$ concentration in different regions due to the COVID-19 pandemic [10–12] . Moreover, various hypotheses have been studied on the relationship between $PM_{2.5}$ and covid-19. Among them, searchers are found that $PM_{2.5}$ has been an important vector in the acceleration of the spread of the COVID-19 [13]. Another paper identifies that a significant relationship between air pollution and COVID-19 infection [14]. The exposures in the long term of $PM_{2.5}$ are positively associated with higher county-level city COVID-19 mortality rates after accounting for many area-level confounders [15]. Based on the hypothesis that there is a relationship between the spread of the virus and the presence of $PM_ {2.5}$ in the air, the researchers propose an innovative metric to predict COVID-19 with the machine learning model quote mirri2020covid.

An effective system for monitoring and predicting air pollution in advance has great importance for human health and government decision-making. However, the mechanism and process of $PM_{2.5}$ formation are very complex due to the complexity of its properties, such as non-linear properties in time and space [16], which have a significant impact on the accuracy of prediction. It thus requires an examining consideration. Furthermore, the air quality data is closely related to time, which means that it belongs to time series and has an apparent periodicity. Due to the data’s timeliness, time predictions have become essential topics that undoubtedly require meticulous attention by academics and scholars. So doing showcases that Time series analysis plays a paramount role in many different applications, including economics, medicine, astronomy, geology, and others.

Traditional statistical methods have been widely used to process air quality forecasting problems. These methods are significantly based on the approach of using historical data for learning. Some of the notable statistical methods that have been used for air quality forecasting are Autoregressive Moving Average ARMA [17], and Autoregressive Integrated Moving Average ARIMA [18]. With the increase in the amount and the complexity of the data obtained, however, these methods can no longer meet the actual demand because of training-length time.

With artificial intelligence and big data evolution, prediction methods based on machine learning technologies are becoming more and more common. Because these types of models do not require an understanding of atmospheric pollutants’ physical or chemical properties. The most popular machine learning algorithms are Multiple Linear Regression (MLR), Random Forest (RF) [19], Support Vector Regression (SVR) [20], Artificial Neural Networks (ANN) [21] that incorporate complex nonlinear relationships between the concentration of air pollutants and meteorological variables. Various ANN structures have been developed to predict air pollution over different study areas, such as neuro-fuzzy neural network (NFNN) [22], and Bayesian neural network [23], An Ensemble Approach which incorporated several different machine learning algorithms, has shown to be a robust and accurate measure of pollution levels in the Greater London area [24].

With the popularity of Artificial Intelligence, many deep learning algorithms have been developed respectively, such as Recurrent Neural Networks (RNN) and their variants. Long short-term memory (LSTM) is the most widely used model in air quality forecasting [25, 26] because it considers the temporal dependencies of a typical phenomenon observed in the $PM_{2.5}$ concentration series. Due to the complexity of $PM_{2.5}$ formation, the high accuracy and demand for predictive efficiency are essential in developing an effective model for predicting $PM_{2.5}$ concentration. We accordingly suggest comparing multivariate deep learning models based on several metrics (Average absolute error MAE,Root mean square error RMSE,The coeffcient of determination $R^{2}$).

To this end, this paper seeks to undergo a research study on the application of deep learning (LSTM, Bi-LSTM, GRU, CNN, CNN-LSTM,CNN-GRU). Hence, the study aims to unearth a comparison between the results obtained with these techniques to learn more about their efficient use in predicting $PM_{2.5}$ concentration. Moreover, our research aims to provide a $PM_{2.5}$ forecasting model with good accuracy with meteorological data and the concentration of adjacent stations.

In this study, we designed a system for the Prediction of $PM_{2.5}$ by utilizing advanced deep neural networks. We, therefore, proposed a hybrid CNN-LSTM forecasting model. Seven baseline predictive deep learning models were also built in this study for comparison with our proposed model. The key contributions of this study are :

1. This study combines the pollutant components, meteorological data, and adjacents stations in different time periods into the input variables. After preprocessing data by filling the missing values, encoding, normalizing data and analyzing the correlation between features and $PM_{2.5}$ concentration as a features selection. Spatial and temporal correlations are complex and comprehensive. In our study, historical data from the target station and adjacent stations are integrated with other features and entered into the model. From the results, the proposed combination is found more effective in extracting spatiotemporal features and performs $PM_{2.5}$ prediction accuracy more than others.
2. Through the proposed model, the Spatio-temporal characteristics of the data are extracted. By combining the advantages of the Convolutional Neural Network CNN model, which is effective at filtering out the spatial characteristics include the characteristics of the data between pollutant components and Weather and between different adjacent stations. At the same time, an LSTM network is used for the extraction of temporal features.
3. Comparing the performances of seven popular deep learning methods in the air pollution prediction problem, we validated the practicality and feasibility of the proposed model in $PM_{2.5}$ concentration prediction by comparing the Metrics in different batch sizes, and lags. Moreover, the results achieved in this work are comparable to other state-of-the-art deep learning approaches reported in the literature.

This paper is organized as follows: “Related works” section briefly reviews the related work. "Deep learning models" section defines the basic concepts of the deep learning models, namely LSTM, Bi-LSTM, GRU, CNN, CNN-LSTM, and CNN-GRU. "Material and methods" section describes the detailed methodology of the proposed approach, including the implementation and experimental results, whereas "Results and discussions" section covers the paper’s conclusion.

## Related works

Since the topic of $PM_{2.5}$ air pollution in cities needs urgently to be solved, $PM_{2.5}$ forecasting is absolutely a vital topic for the development of smart cities. The difficulty of prediction can be seen in the fact that $PM_{2.5}$ propagation is impacted by variations in meteorological variables, e.g. Wind speed and direction. Wind speed and direction data have a high degree of randomness and constantly change over different periods [27, 28].

Several $PM_{2.5}$ prediction methods are developed by researchers based on statistical models and machine learning techniques. Recently, the academic community has begun using deep neural networks for pollutant concentration prediction. Deep learning may solve problems by using more layers and more extensive data sets and processing all layers simultaneously to obtain more accurate results [29]. These favorable properties of deep learning make it suitable for modeling and predicting air pollution.

A wide variety of models can be used for this purpose. Authors in article [30] analyse and study the prediction $PM_{2.5}$ levels on 12 stations in Beijing using four models ARIMA, FBProphet (Facebook prophet), LSTM, and CNN. With historical air quality data, meteorological data, weather forecast data. Most of the stations showed that LSTM performed better than all other models MAE = 13.2 and RMSE = 20.8. In this study [31], the authors propose a predictive model of PM concentration at the 25 monitoring stations in Seoul, South Korea, historical PM2.5 concentration, and meteorological data is used for comparing LSTM, and DAE (Denoising AutoEncoders). The comparison showed that the LSTM prediction model was more accurate than the DAE model.

In article [32] , the authors develop a bidirectional long short-term memory (Bi-LSTM) model to predicted $PM_{2.5}$ Concentration in China. The $PM_{2.5}$ Concentration and weather from the hourly data of the US Embassy, recorded for Beijing city as input. The proposed model achieved accuracy as follows MAE = 7.53, RMSE = 9.86, and SMAPE = 0.1664. Other researchers have been interested to Predict the $PM_{2.5}$ contamination of stations in Beijing by using long short-term memory-fully connected (LSTM-FC), LSTM, and an artificial neural network (ANN) with historical air quality data, meteorological data, weather forecast data, and the day of the week data. They showed that the LSTM-FC model outperforms LSTM and the ANN, with MAE = 23.97 and RMSE = 35.82 over 1-6 h [33]. However, none of these models can make use of pollutant concentration information in neighboring areas. Changes in pollutants are related not just to time but also to space. Because a pollutant in one place may travel to other regions, spatial information must be considered.

A CNN is consisting of a series of convolutional layers used to extract the spatial features of neural networks. CNN achieved remarkable results in multi-dimensional spatial arrays Which makes it a good topic for researchers to know the environmental situation through digital images. In the article [34], the authors propose an ensemble of deep neural networks to estimate $PM_{2.5}$ concentrations from outdoor images. Three convolutional neural networks, VGG-16, Inception-v3, and Resnet50, are used as the base learners. The experimental results demonstrated that the proposed ensemble can provide a more accurate $PM_{2.5}$ estimation than all three individual deep learning networks used. CNN has proven to be powerful in spatial data processing. This method has also been used to estimate the concentration of pollutants in urban areas, usually by analyzing satellite images [35, 36]. However, sometimes there is no image data but only abstracted monitoring data, e.g., wind direction, temperature, and location.

To solve the problem of Air Pollution in Seoul city in Korea, the researchers proposed the usage of the Convolutional Long Short-Term Memory (ConvLSTM), a combination of Convolutional Neural Networks and Long Short-Term Memory, which automatically manipulates both the spatial and temporal features of the data [37]. In this paper, this Spatio-temporal model includes air pollution data, meteorological data, traffic volume, average driving speed, and air pollution indicators in outdoor areas. The proposed model has proven its superiority over the various models. In another paper [38], the authors verified the feasibility and practicability of CNN-LSTM to estimate $PM_{2.5}$ concentration in Beijing for the next hour, cumulated wind speed, and cumulated hours of rain over the last 24 h. They showed that the CNN-LSTM model outperforms other models with MAE = 14.6344 and RMSE = 24.22874.

## Deep learning models

In this work, our goal is to investigate the performances of several deep learning models to forecast the concentration of $PM_{2.5}$. Thus, we decided to use the LSTM, Bi-LSTM, GRU, CNN, CNN-LSTM previously mentioned. Next, we briefly describe each network:

### LSTM

LSTM is a type of recurrent neural network (RNN) that was developed in 1980 [39, 40]. RNNs are a powerful type of artificial neural network and are most likely used for time-series forecasting problems. RNN can internally maintain memory to remember things from past occurrences that can predict future events. However, RNNs frequently suffer from vanishing and exploding gradients, which leads the model learning to become too slow or stopped altogether. LSTMs were created in 1997 [41] to solve these problems. LSTMs have longer memories and can learn from inputs that are separated from each other by long time lags.

**Fig. 1:**
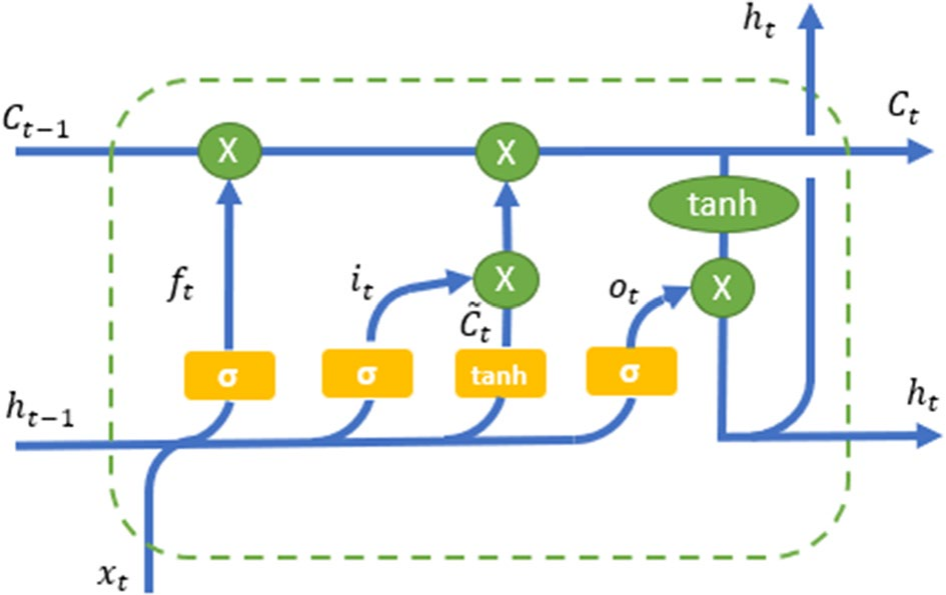
Architecture of the LSTM cell

An LSTM has three gates: an input gate that determines whether or not to let the new input in, a forget gate that deletes information that is not important and an output gate that decides what information to output. These three gates are analogical gates based on the sigmoid function, which works on the range between 0 and 1. These three sigmoid gates can be seen in Fig. 1 below. A horizontal line that can be seen running through the cell represents the cell state.

LSTM formulas are listed below:

1 $$\begin{aligned}&Input gate \qquad : \qquad i_{t}=\sigma (W^{(it)}{\overline{x}}_{t}+W^{(it)}{h}_{t-1}) \end{aligned}$$

2 $$\begin{aligned}&Forget gate \qquad : \qquad f_{t}=\sigma (W^{(if)}{\overline{x}}_{t}+W^{(hf)}{h}_{t-1}) \end{aligned}$$

3 $$\begin{aligned}&Output gate \qquad : \qquad i_{t}=\sigma (W^{(io)}{\overline{x}}_{t}+W^{(ho)}{h}_{t-1}) \end{aligned}$$

4 $$\begin{aligned}&Process Input \quad : {\widetilde{C}}_{t}=tanh(W^{(i{\widetilde{c}})}{\overline{x}}_{t}+W^{(h{\widetilde{c}})}{h}_{t-1}) \end{aligned}$$

5 $$\begin{aligned}&Cell update \qquad : \qquad {C}_{t}=f_{t}*C_{t-1}+i_{t}*{\widetilde{C}}_{t} \end{aligned}$$

6 $$\begin{aligned}&Output \qquad  : \qquad {y}_{t}=h_{t}=O_{t}+i_{t}*tanh({C}_{t}) \end{aligned}$$

### GRU

**Fig. 2:**
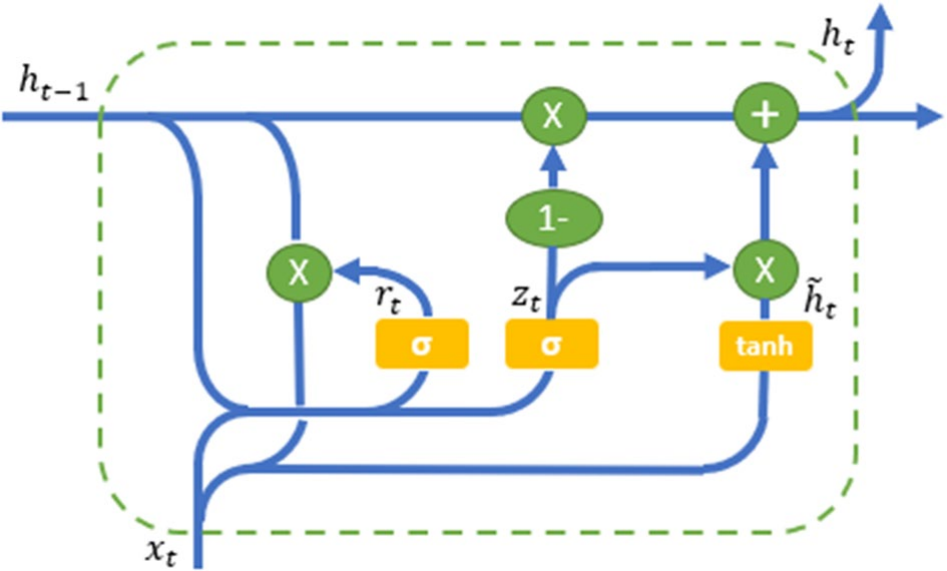
Architecture of the GRU cell

GRU, Gated recurrent unit is an advancement of the standard RNN [33] is included in RNN, and it is similar to an LSTM unit. The GRU unit consists of the reset and updates gate. Figure 2 shows the GRU architecture. The reset gate is designed to forget the previous state between the prior activation and the next candidate activation, whereas the update gate is used to select the number of the candidate activation that updates the cell state.

GRU formulas are listed below:

7 $$\begin{aligned}&Reset gate \qquad  : \qquad r_{t}=\sigma (W^{(ir)}{\overline{x}}_{t}+W^{(hr)}{h}_{t-1}) \qquad \end{aligned}$$

8 $$\begin{aligned}&Update gate \qquad  : \qquad z_{t}=\sigma (W^{(iz)}{\overline{x}}_{t}+W^{(hz)}{h}_{t-1}) \qquad \end{aligned}$$

9 $$\begin{aligned} Process Input \qquad : \qquad {\widetilde{h}}_{t}=tanh(W^{(i{\widetilde{h}})}{\overline{x}}_{t}+W^{(h{\widetilde{h}})}{h}_{t-1}) \end{aligned}$$

10 $$\begin{aligned}Hidden state update \qquad  : \qquad h_{t}=(1-z_{t})*h_{t-1}+z_{t}*{\widetilde{h}}_{t} \qquad \end{aligned}$$

11 $$\begin{aligned}Output \qquad \qquad \qquad : \qquad y_{t}=h_{t} \qquad \qquad \quad \qquad \qquad \qquad \end{aligned}$$

### Bi-LSTM

**Fig. 3:**
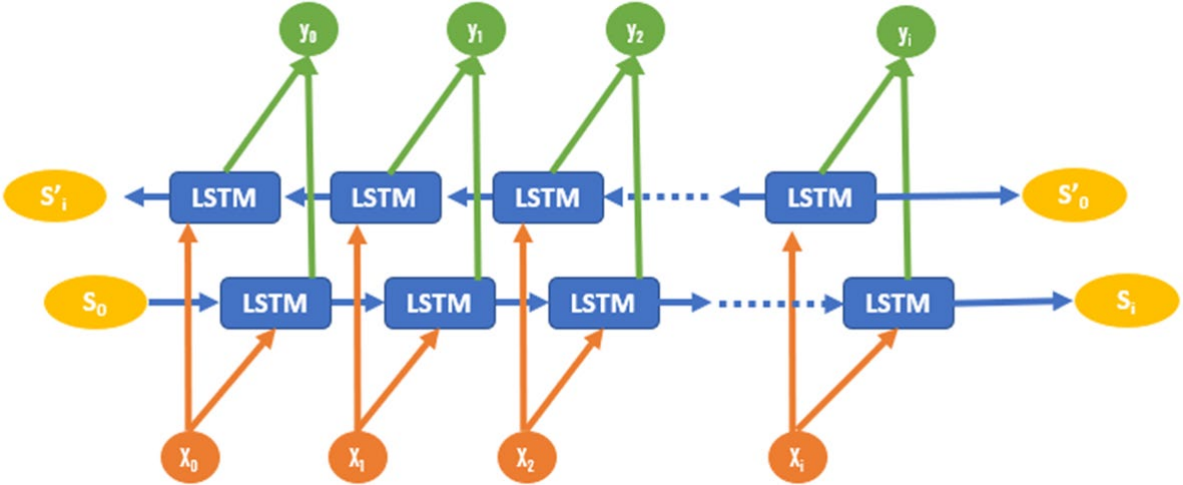
Architecture of the Bi-LSTM model

Standard RNN and LSTM often ignore future information in time-processing, while Bi-LSTM can take advantage of future information. The basic structural idea of Bi-LSTM is that the front and back layers of each training sequence are two LSTM networks, respectively. Moreover, the LSTM networks are both connected to one input and one output layer. The output layer can obtain past information of each point from the input sequence and get future information from each point through this structure. as shown in Fig. 3.

### CNN

**Fig. 4:**
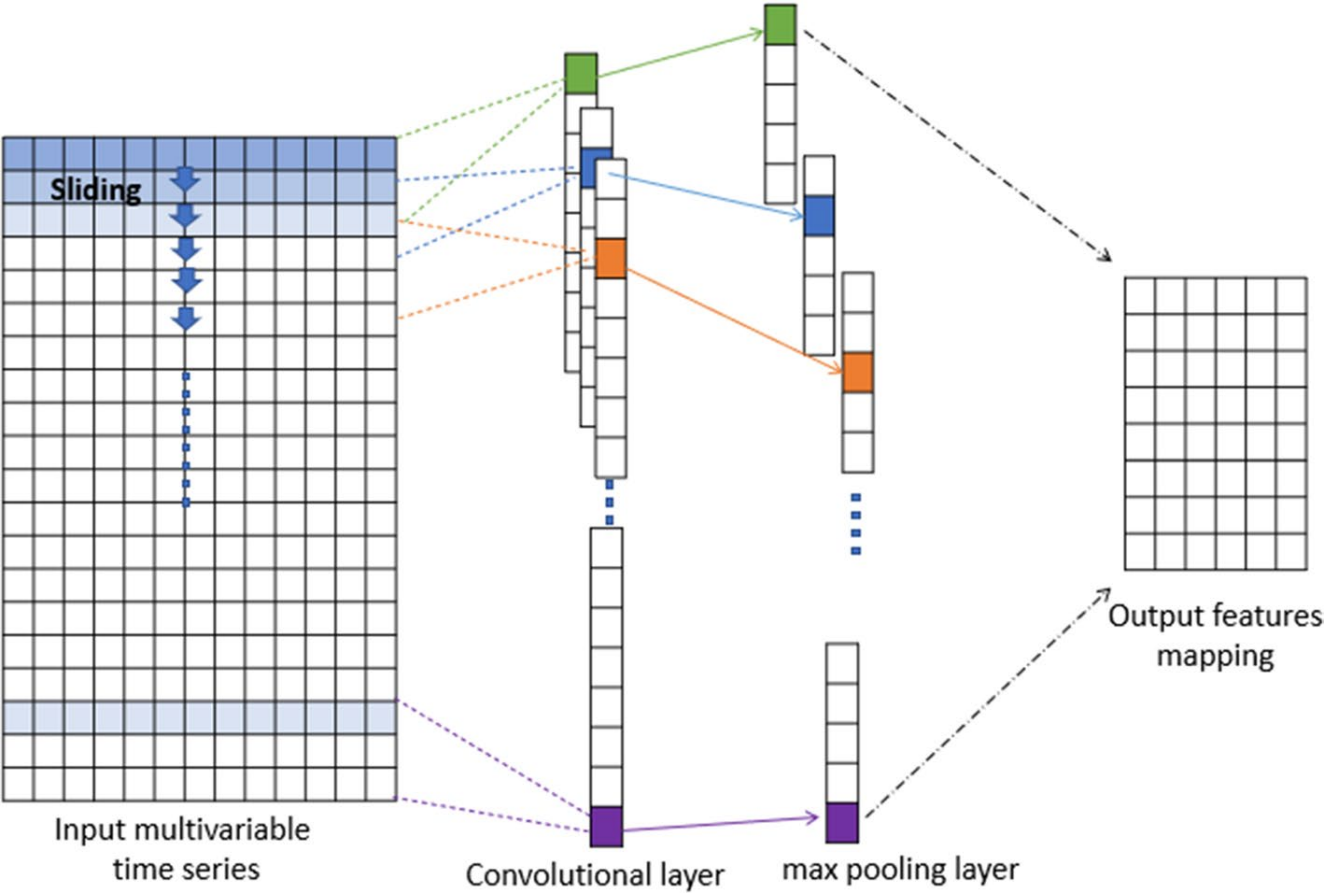
Architecture of the CNN model

CNN has been successfully applied to computer vision and medical image analysis [42]. Moreover, in this paper auteurs proposes a multiscale fully convolutional neural network (MFCN) for change detection in high-resolution remote sensing images [43]. In our model, the convolutional layers are constructed using one-dimensional kernels that move through the sequence (unlike images where 2D convolutions are utilized). These kernels act as filters that are learned during training. As in many CNN architectures, the deeper the layers get, the higher the number of filters. The architecture of CNN is shown in Fig. 4.

### CNN-LSTM

The use of classical CNN architecture is the best choice when input networks are 2-D or 3-D tensors like images or videos [44]. Since LSTMs architectures are more adapted for 1-D Data, a new variant of LSTM called Convolutional LSTM or ConvLSTM [45] has been designed. In this architecture, the LSTM cell, which contains a convolution operation and input dimension of data, is kept in the output layer instead of just a 1-D vector. A convolution operation replaces matrix multiplication at each gate of classical LSTM. We can say that ConvLSTM architecture merges the capabilities of CNN and LSTM Network. It was normally developed for 2-D Spatio-temporal data such as satellite images.

**Fig. 5:**
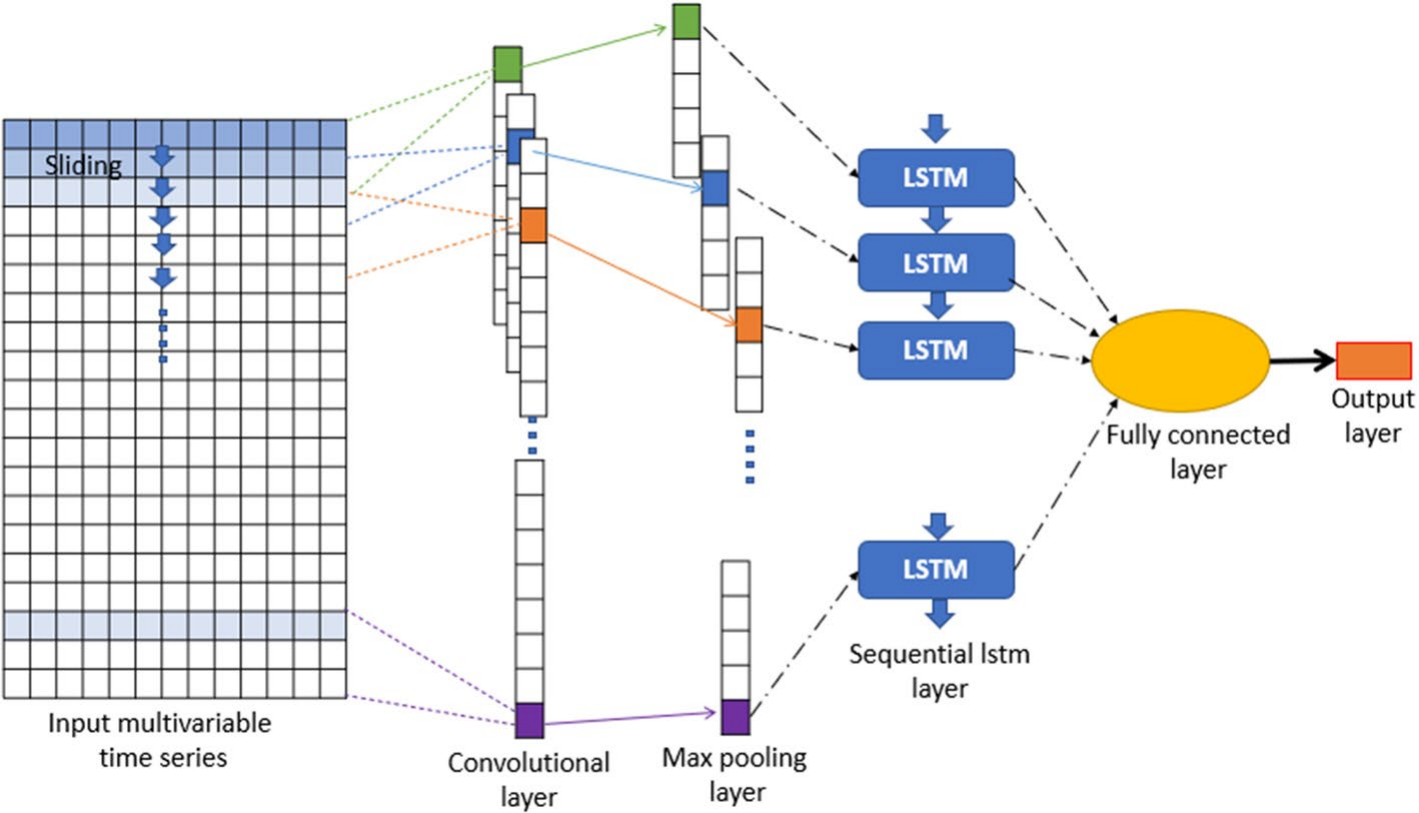
Architecture of the CNN-LSTM Model

Another approach to working with Spatio-temporal data is to combine CNN and LSTM layers, one block after another. Such architecture is called Convolutional-LSTM (CNN-LSTM) and was initially named Long-term Recurrent Convolutional Network or LRCN model. In the first part of this model, convolutional layers extract essential features of input data, and the results are flattened in a 1-D tensor so that they can be used as input for the second part of the model (LSTM). Finally, before passing data in the last hidden layer, information has to be reshaped in the original form of input data. The architecture of CNN-LSTM is shown in Fig. 5.

## Material and methods

### Dataset

**Fig. 6:**
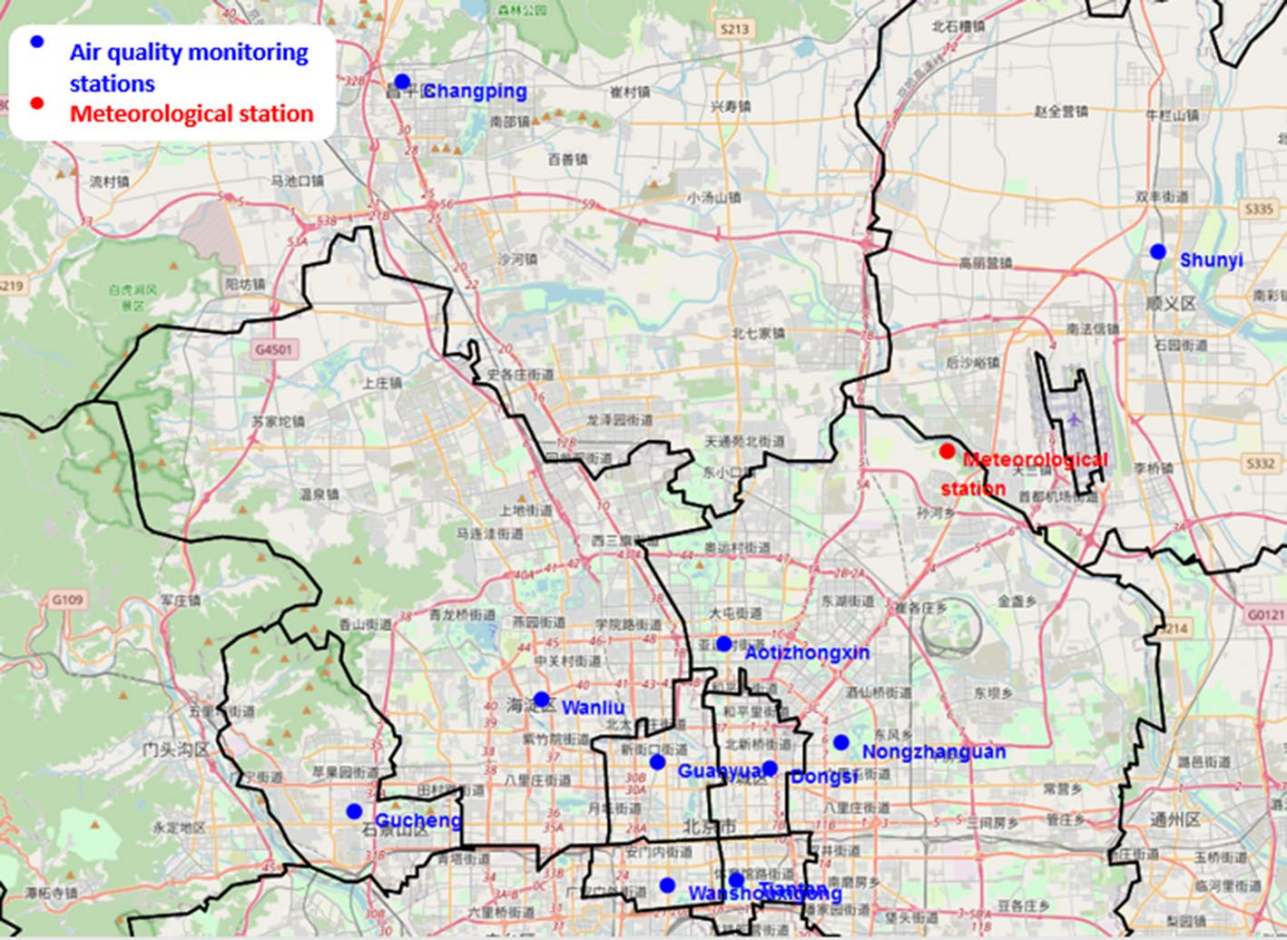
The distribution of monitoring stations in Beijing

The dataset chosen in this article (420768 instances and 18 attributes) comes from the UCI Machine Learning Repository [46]. this dataset shows the concentration of air pollutants and air quality at 12 sites. The air quality data comes from the Beijing Municipal Environmental Monitoring Center. The meteorological data indicating the air quality for each site is matched with the nearest meteorological station of the China Meteorological Administration. as shown in Fig. 6.

### Data pre-processing

#### Missing values

This dataset includes 35064 records with multi-features in each station. The period of recording is from March 1st, 2013, to February 28th, 2017. The data are composed of: date, the concentration of $PM_{2.5}$, $PM_{10}$, Sulfur dioxide $SO_{2}$, Nitrogen dioxide $NO_{2}$, carbon monoxide *CO*, ozone $O_{3}$, dew point, temperature, atmospheric pressure, combined wind direction, cumulated wind speed, cumulated hours of snow, and rain. However, Air quality and meteorological monitoring equipment will cause leakage in data collection due to machine failure, due to some uncontrollable reasons. The existence of such missing values will have some impact on data mining.

In time-independent (non-chronological) data to replace missing field values, the most popular approaches are the mean or median value. However, in the case of a time series, this is not the case. To resolve incomplete data problems, many imputation techniques are adopted. A study has shown that the linear interpolation method is the best method to estimate hourly monitoring data for $PM_ {10}$ for all percentages of simulated missing values [47].

The processed data set contains less than 4% missing values, these missing values were addressed by linear spline imputation. The *SL*(*x*) equation can adapt to local anomalies without affecting the interpolation values at other points.

The equation of the spline linear interpolation function is:

12 $$\begin{aligned} \quad SL(x)=f(x_{i-1})\frac{x-x_{i}}{x_{i-1}-x_{i}}+f(x_{i})\frac{x-x_{i-1}}{x_{i}-x_{i-1}} \quad x\in [x_{i-1},x_{i}], i=1,2,3,...,n \end{aligned}$$

where *x* is the independent variable, $x_{0}$, $x_{1}$, ... $x_{n}$ are known values of the spline and *SL*(*x*) the linear spline that interpolates *f* at these points.

#### Encoding categorical variables

**Fig. 7:**
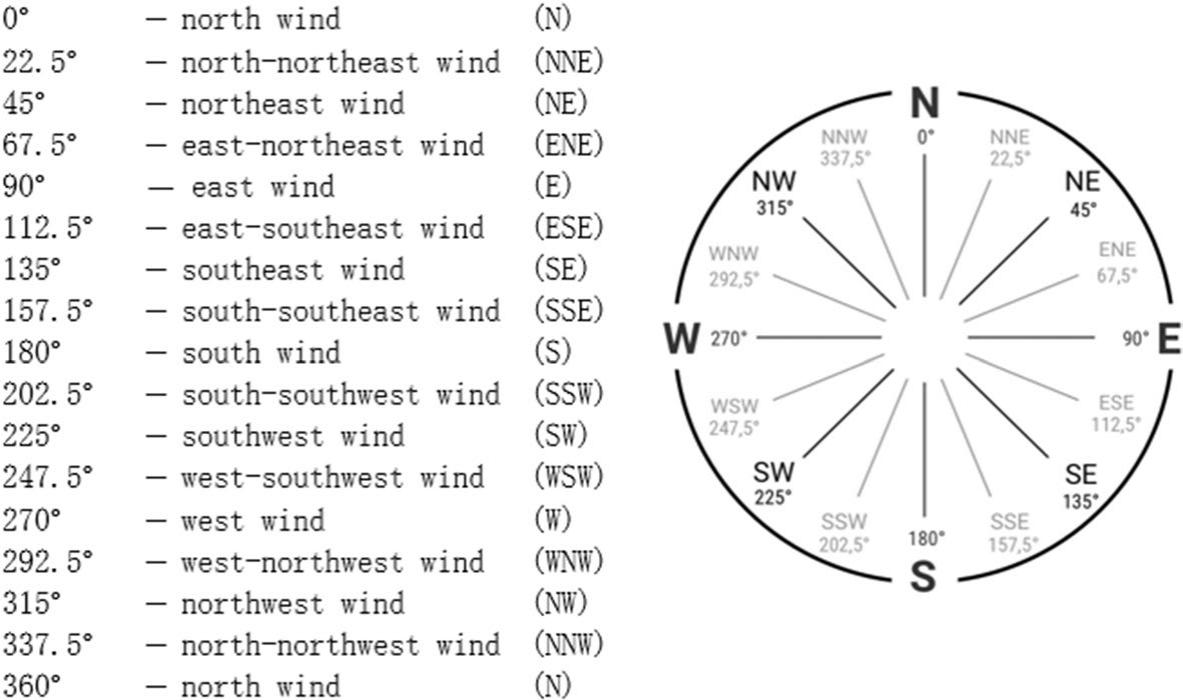
Wind Direction and Degree Values

In this analysis, the wind factor is an essential indicator of atmospheric activity. The pollutant concentration is affected by the wind speed [27], and the wind direction is crucial in determining the concentrations of $PM_ {2.5}$ [28]. The wind direction attribute is categorical data, admits 16 values: N, NNE, NE, ENE, E, ESE, SE, SSE, S, SSW, SW, WSW, W, WNW, NW, and NNW. To convert each cardinal wind direction to a value of degrees azimuth. We have divided the compass into 16 sectors of 22.5 degrees each. North was given a value of zero and with clockwise displacement, the value increase by 22.5. The direction of each segment is 22.5 degrees. as shown in Fig. 7.

#### Normalization

In order to improve the prediction accuracy, we normalize the values of $PM_{2.5}$ concentration using the Min-Max normalization, the method is given in the equation 13:

13 $$\begin{aligned} \qquad \qquad \qquad \qquad \qquad x=\frac{x-min}{max-min} \end{aligned}$$

### Feature selection

In machine learning applications, features selection is an essential step that can be done in several ways. Most of the previous work has applied a mathematical correlation to find the relationship between the input and output variables [48–51]. When there are many features to enter the network for training, finding the correlation between the target output value and those features reduces the complexity of training and improves performance [48].

The Pearson correlation is the most popular method used to find the correlation between two variables. The following equation can calculate its coefficient *r*:

14 $$\begin{aligned} \qquad \qquad \qquad \qquad r=\frac{\sum _{i=1}^{n}(x_{i}-{\bar{x}})(y_{i}-{\bar{y}})}{\sqrt{^{\sum _{i=1}^{n}(x_{i}-{\bar{x}})^{2}}}\sqrt{^{\sum _{i=1}^{n}(y_{i}-{\bar{y}})^{2}}}} \end{aligned}$$

where *x* and *y* represent variables, and ${\bar{x}}$ and ${\bar{y}}$ represent the mean of the variables.

#### Air quality feature

**Fig. 8:**
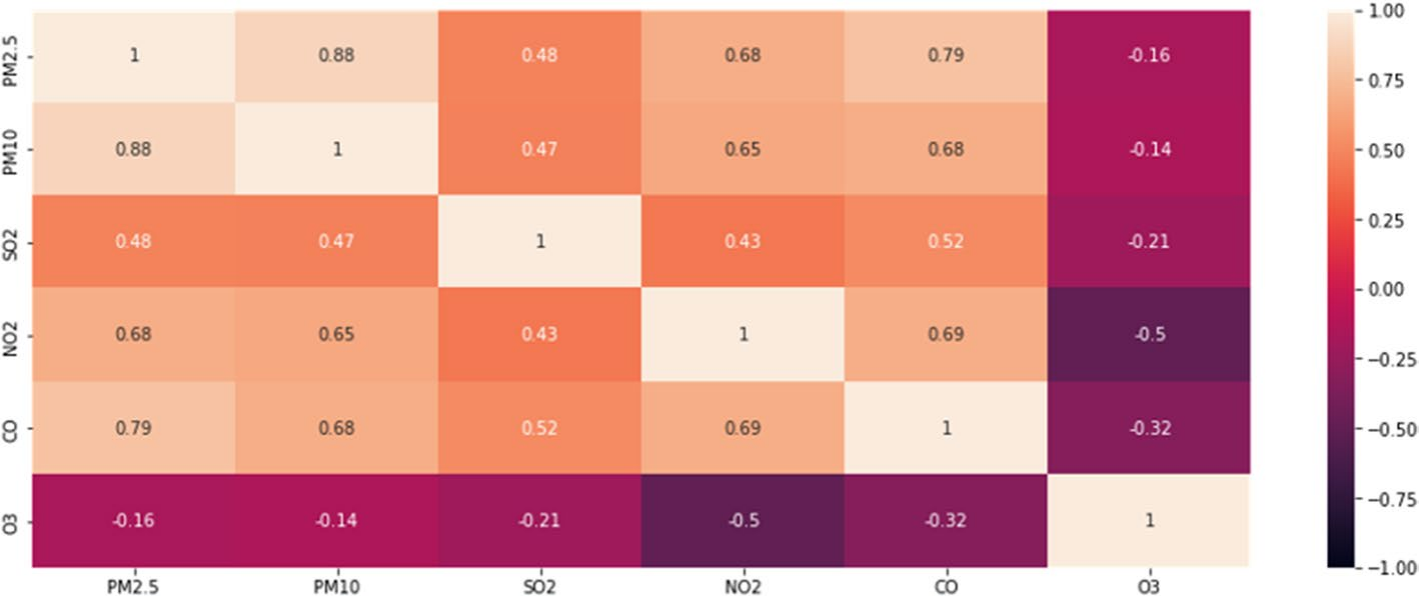
The correlation matrix of the air quality features

In the atmosphere, we detect different pollutants, the increase of their concentrations negatively affects the quality of the air. We calculated the correlations between the features, of the air quality and we found a high correlation value between $PM_{2.5}$, $PM_{10}$, and *CO* as shown in Fig. 8.

#### Meteorological feature

**Fig. 9:**
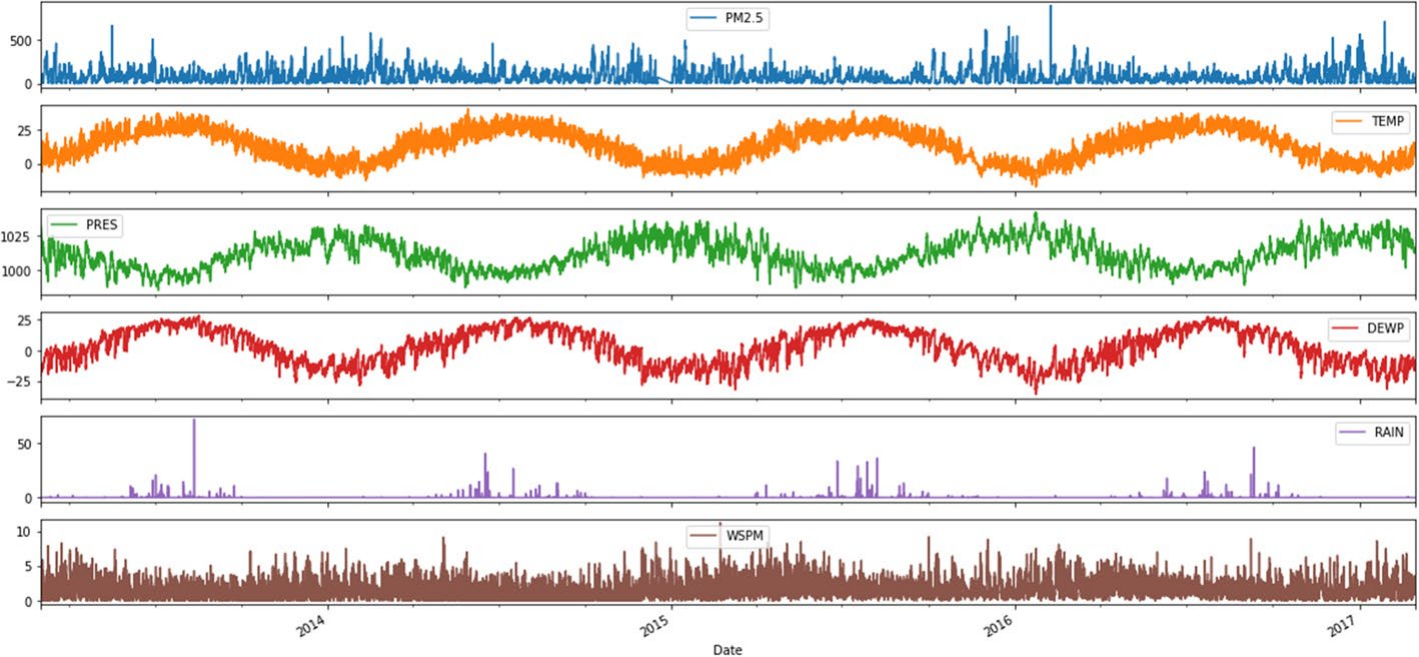
Graphical representation of Meteorological data and $PM_{2.5}$

Weather parameters (atmospheric temperature, atmospheric pressure, wind speed, wind direction, and relative humidity) affect air quality. For example, high wind speed will reduce the concentration of $PM_{2.5}$, high humidity generally worsens air pollution, and high air pressure generally results in good air quality [50, 51]. Therefore, meteorological parameters are of prime importance for the task of forecasting air quality (Fig. 9).

#### Spatial analysis

We performed the spatial correlation between Aotizhongxin station (target) and other adjacent stations. We used Pearson correlation to select the correlated $PM_ {2.5}$ monitoring stations around the target.

**Fig. 10:**
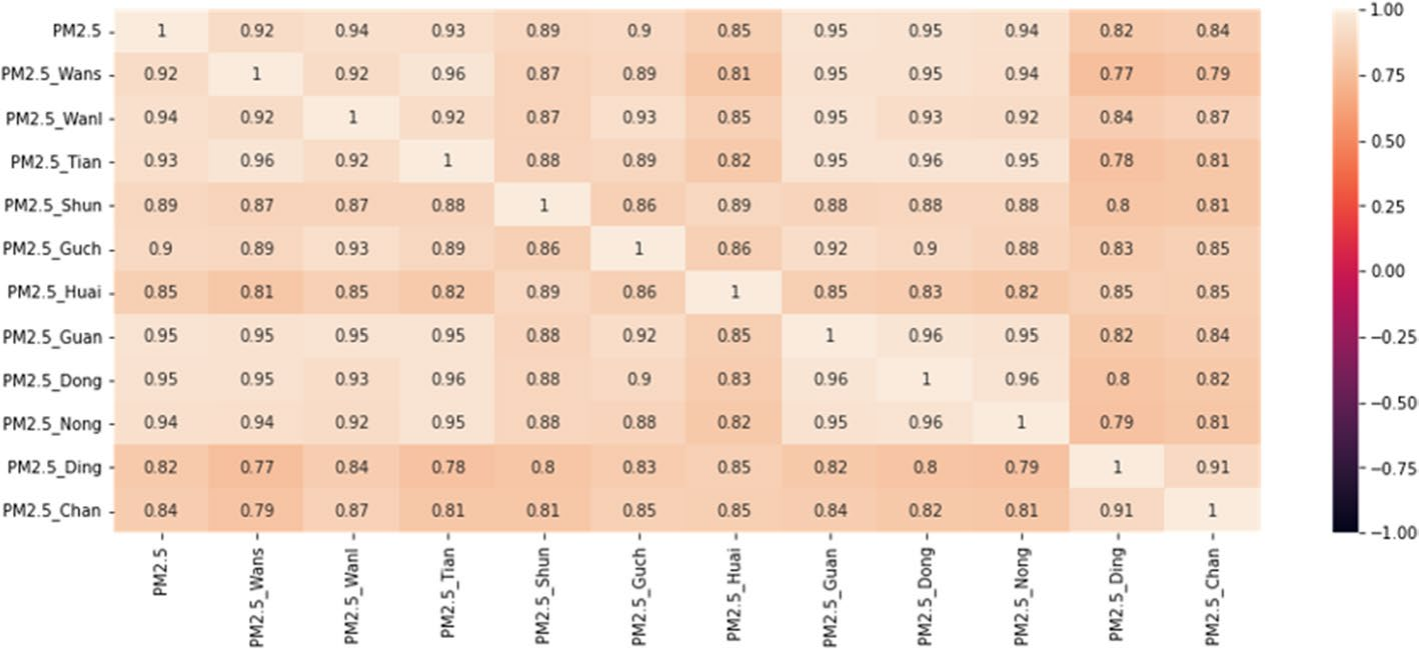
The Spatiotemporal Correlation Analysis

The results are shown in Fig. 10. All correlation values are above 0.80 indicate that there is a strong spatial correlation between the selected stations.

The data set has been split into two, a training set and a test set. 80% (28,052 h) of the dataset was taken as a training set. The remaining 20% (7012 h) becomes the test set used to test the model and analyze its accuracy.

### Evaluation index of the models

Once the structure of the model is determined, the training set is used to train the network until convergence. In order to assess the efficiency of the model, three indicators are used in this article, including the mean absolute error (MAE), the mean squared error (RMSE), and the coefficient of determination ($R^{2}$).

#### MAE

MAE (Mean Absolute Error) is the arithmetic mean of the absolute values of the deviations between the true value and the model prediction value of all samples, which can better reflect the real prediction error situation. The calculation formula is as follows:

15 $$\begin{aligned} \qquad \qquad \qquad \qquad MAE=\frac{1}{n}\sum _{i=1}^{n}\left| y_{i}-{\hat{y}}_{i}\right| \end{aligned}$$

#### RMSE

RMSE (Root Mean Square Error) is the square root of the mean of the square of all of the error. It may well reflect the accuracy of the prediction error. The calculation formula is shown below:

16 $$\begin{aligned} \qquad \qquad \qquad \qquad RMSE=\sqrt{\frac{1}{n}\sum _{i=1}^{n}( y_{i}-{\hat{y}}_{i})^{2}} \end{aligned}$$

#### $R^{2}$

The coefficient of determination reflects the proportion of all variations of the dependent variable that can be explained by the independent variable through the regression relationship. The closer the value of $R^{2}$ is to 1 becomes, the better the independent variable can explain the dependent variable. See the calculation formula below:

17 $$\begin{aligned} \qquad \qquad \qquad \qquad R^{2}=\frac{\sum _{i=1}^{n}( y_{i}-{\hat{y}}_{i})^{2}}{\sum _{i=1}^{n}( y_{i}-{\bar{y}}_{i})^{2}} \end{aligned}$$

In these three equations, *n* is the sample size, $y_{i}$ and ${\hat{y}}_{i}$ represent the real value and predicted value at time, respectively; ${\bar{y}}_{i}$ denotes the mean of all real values.

## Results and discussions

We designed our models with various Python packages, including Scikit-Learn, Keras, and native TensorFlow. For hardware, We ran our heavier workloads on Google Colab, which housed NVIDIA’s Tesla T4 GPU.

**Fig. 11:**
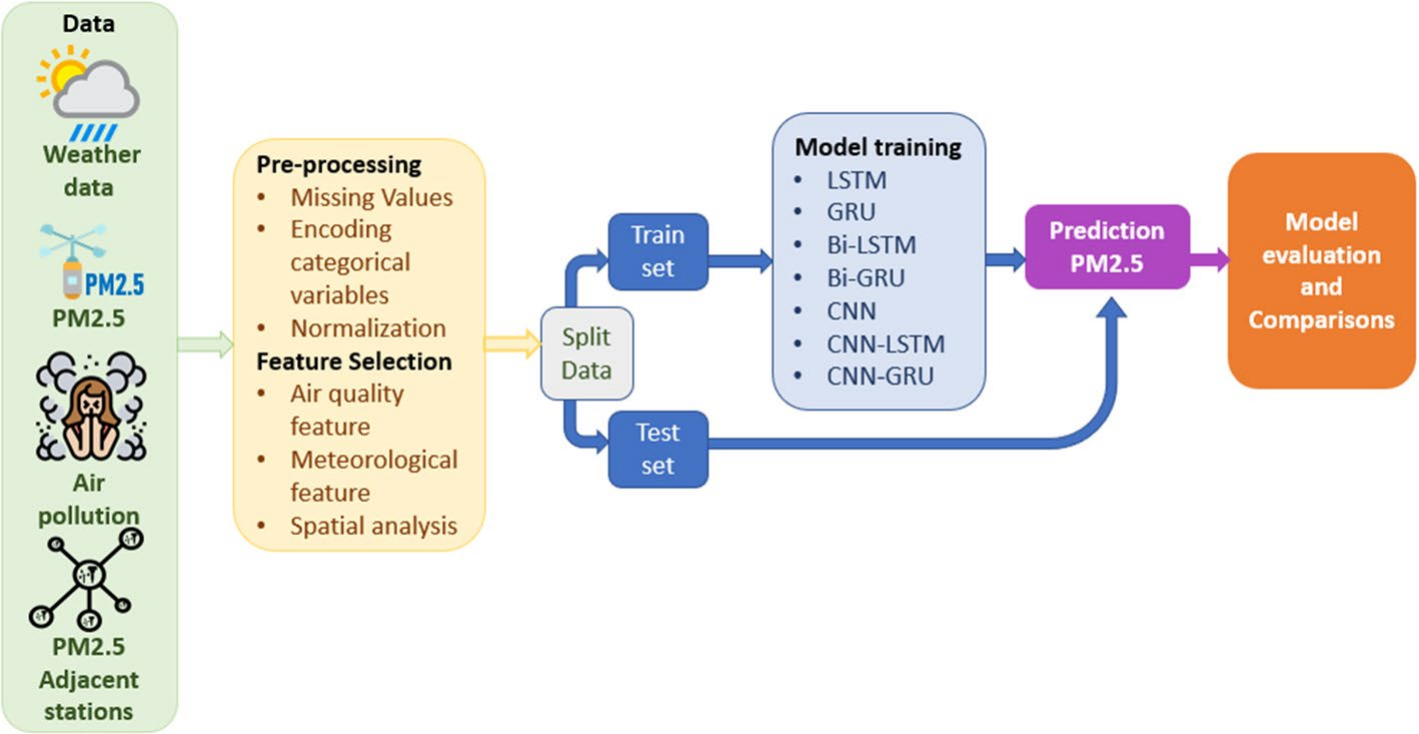
Workflow for predicting $PM_{2.5}$ concentrations

In this research, the prediction of the concentration of $PM_{2.5}$ was simulated using various deep-learning models. In this section, the historical observation $PM_{2.5}$ data are compared with the computed $PM_{2.5}$ from artificial neural networks such as LSTM, GRU, Bi-LSTM, Bi-GRU, CNN, CNN-LSTM, and CNN-GRU tested in one and seven lag days. Figure 11 shows the workflow for predicting $PM_{2.5}$ concentrations.

Each network attempts to predict the results as accurately as possible. The value of the accuracy in the network is achieved by the cost function trying to punish the network when it fails. The optimal output is the lowest cost.

In this study, for all networks, we applied MSE (Mean Squared Error) as a cost function. A repetition step in training generally works with a division of training data named a batch size. The number of samples for each batch is a hyperparameter, generally obtained by trial and error.

The value of this parameter in all models is 24, 32, 64, and 128, respectively, as this study has shown. In each repetition step, the cost function is computed as the mean MSE of these observed and predicted $PM_{2.5}$ concentration samples. The number of iteration steps for neural networks is named an epoch; in each epoch, the streamflow time series is simulated by the network once. Like other networks, neurons or network layers can be selected arbitrarily in recurrent networks. In our study for comparing models with each other, the structures of all recurrent network models are created identically.

**Fig. 12:**
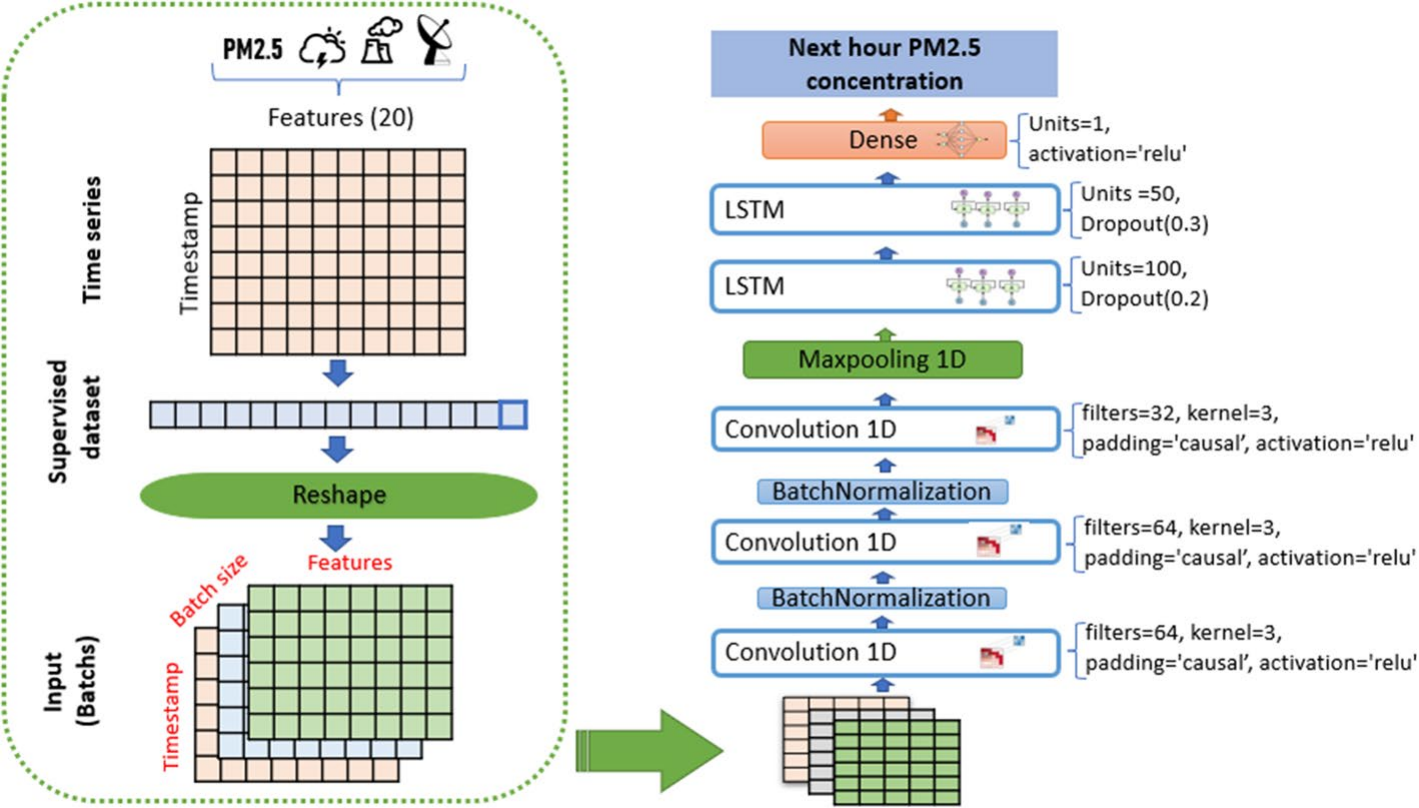
The architecture of the proposed CNN-LSTM

- In LSTM, GRU, BI-LSTM, and BI-GRU each network, four hidden layers are used, 200 units in the first layer, then 100 in the second layer, and 50 units in the last two layers. The last layer output of the network is linked to a dense layer with a single output neuron. Between the layers, a dropout equal to 10% is used. In all networks, the ReLU [52] activation function is applied for the hidden layers.
- In CNN-LSTM and CNN-GRU each network of them contains 1D CNN, which Contains three convolutional layers, with 64, 64, and 32 feature detectors successively, the length of the convolution window is 3 with causal padding. Between the three convolutional layers, the BatchNormalization layer is used. All is followed by a MaxPooling1D layer with a pool size of 3. This last is linked to LSTM/GRU, which Contains two layers with 100 and 50 units per layer, then a dense layer with a single output neuron. An overview of the proposed CNN–LSTM models architecture is depicted in Fig. 12.

The main advantage of using ReLU is that there is a fixed derivative for all inputs greater than 0. This constant derivative speeds up network learning. Each method is run with 200 epoch, and a $EarlyStopping (min\_delta=1e-3, patience=50)$. All models are run with different Batch sizes. As seen in Table 1, the Batch size as one of the influential parameters plays a primary role. We used Adam as an optimizer with the learning rate ( 0,001) and learning rate decay (0.0001). As showcased in Table 1, three different evaluation criteria compare seven different prediction methods.

**Table 1:** Results of different models in 1 and 7-days Lags (Bold indicates the best results.)

Model	Batch	1 Day			7 Day		
		MAE	RMSE	$R^{2}$	MAE	RMSE	$R^{2}$
GRU	**24**	9.960	16.571	0.979	12.868	21.526	0.953
	**32**	9.541	16.609	0.978	11.055	18.122	0.974
	**64**	10.021	17.220	0.977	12.258	19.797	0.965
	**128**	9.842	16.904	0.978	11.899	19.560	0.970
LSTM	**24**	9.102	15.999	0.980	11.916	19.255	0.969
	**32**	9.503	16.217	0.980	11.623	19.154	0.972
	**64**	9.252	16.044	0.980	11.551	19.155	0.970
	**128**	9.725	16.997	0.978	11.823	19.227	0.971
Bi-LSTM	**24**	8.947	15.710	0.982	12.204	20.050	0.966
	**32**	8.868	15.597	0.982	11.253	18.488	0.974
	**64**	9.561	16.380	0.980	12.055	19.323	0.969
	**128**	9.488	16.456	0.979	11.753	18.113	0.971
Bi-GRU	**24**	9.907	16.859	0.978	11.488	18.854	0.969
	**32**	9.692	16.712	0.978	11.984	19.294	0.970
	**64**	9.192	16.196	0.981	11.631	19.289	0.969
	**128**	9.230	16.046	0.981	11.553	19.113	0.970
CNN	**24**	9.663	17.062	0.978	10.693	17.962	0.973
	**32**	9.591	16.981	0.979	11.150	18.606	0.975
	**64**	9.261	16.667	0.979	10.621	18.431	0.975
	**128**	9.974	17.636	0.977	10.674	18.636	0.974
CNN-LSTM	**24**	9.198	16.523	0.981	9.353	16.724	0.978
	**32**	**6**.**742**	**12**.**921**	**0**.**989**	**9**.**034**	**16**.**625**	**0**.**979**
	**64**	7.869	15.757	0.982	9.885	18.373	0.976
	**128**	8.940	16.337	0.980	9.037	16.524	0.979
CNN-GRU	**24**	9.812	17.554	0.977	9.912	18.564	0.965
	**32**	9.459	17.700	0.977	9.459	18.700	0.967
	**64**	9.433	16.836	0.979	9.653	17.856	0.976
	**128**	9.499	17.285	0.979	9.949	17.885	0.976

Table 1 summarizes the MAE, RMSE, and $R^{2}$ values for the concentration of $PM_{2.5}$ in air generated by the model prediction models. In 65 models, the RMSE values for the 1-day lags were the smallest. However, the results show that the CNN-LSTM performed best in a one-hour forecast compared to other models under the same conditions, and different batches sizes. Moreover, these results show that the CNN-LSTM with 32 in batch size is more accurate in the different lags, with an advantage in 1-day lag.

**Fig. 13:**
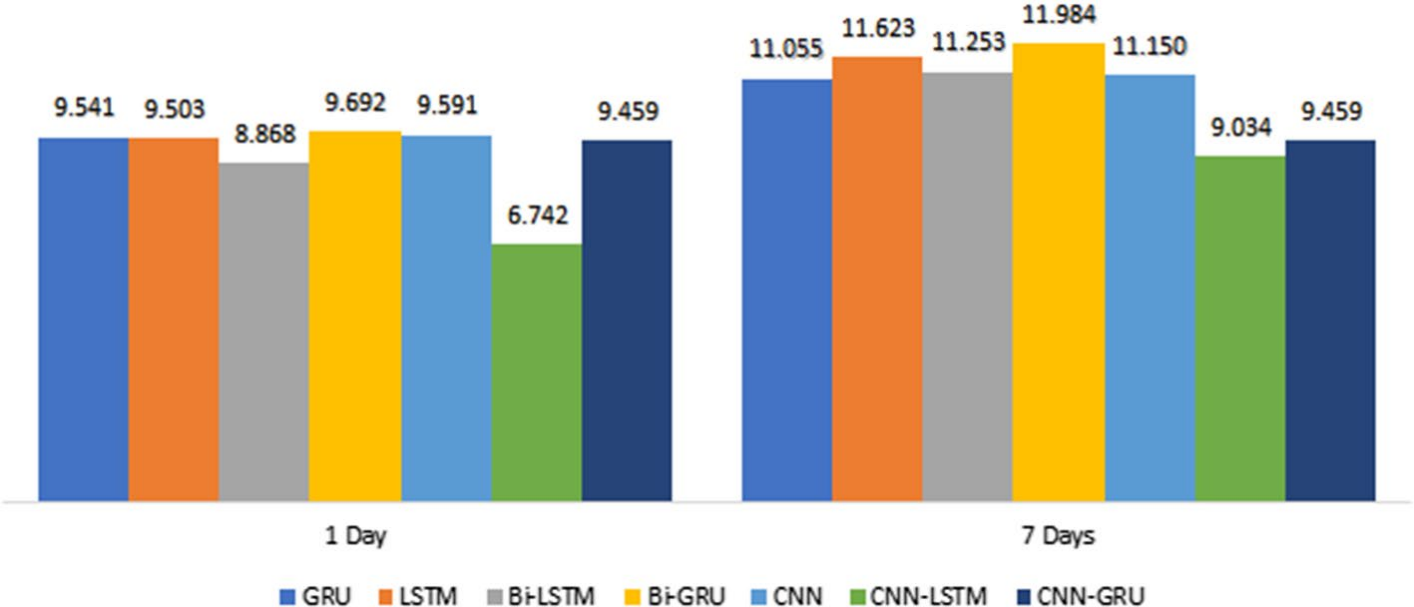
Comparison of the MAE for the 1day and 7day lag for the different deep learning models

**Fig. 14:**
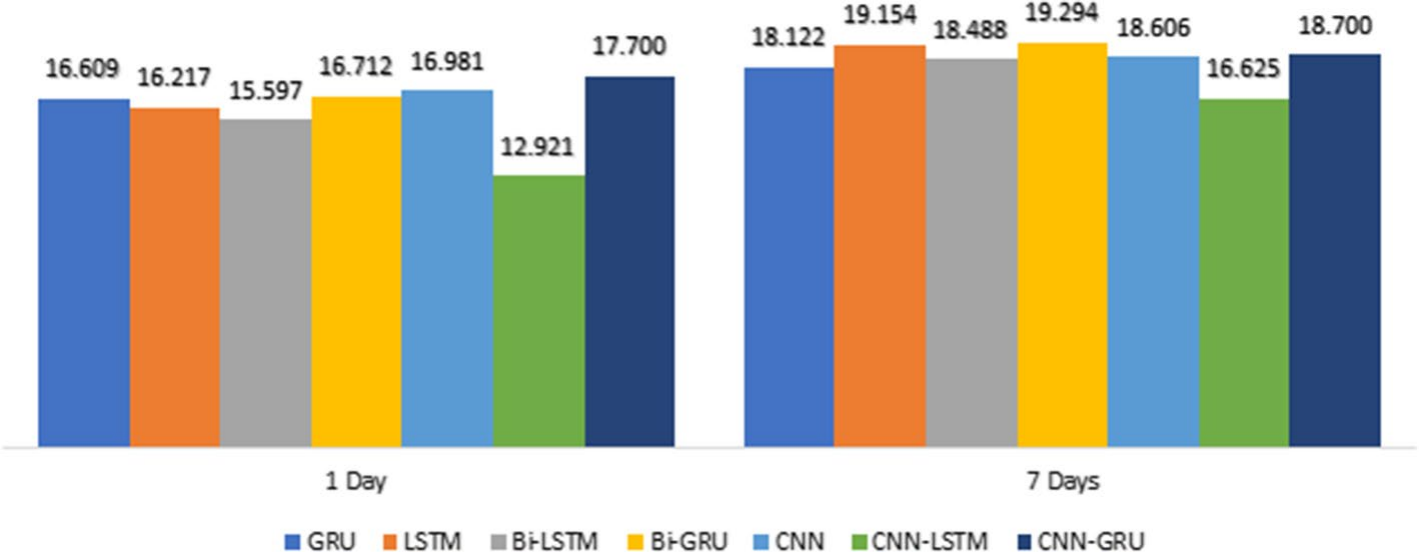
Comparison of the RMSE for the 1day and 7day lag for the different deep learning models

**Fig. 15:**
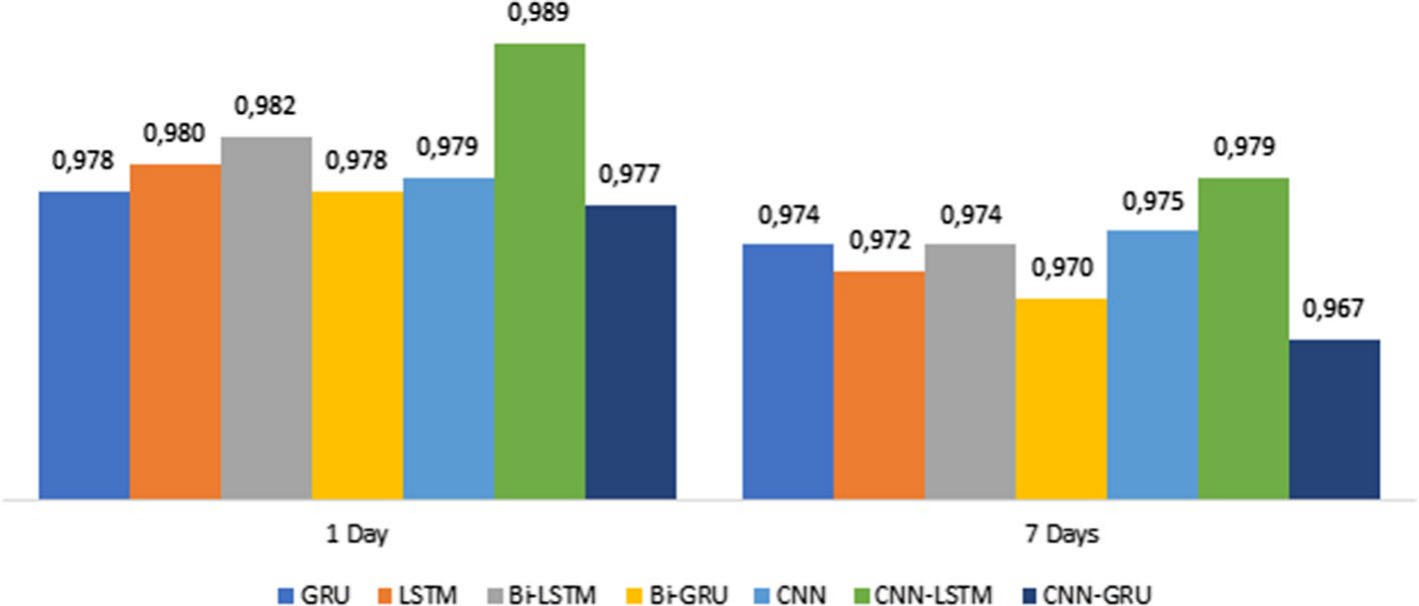
Comparison of the $R^{2}$ for the 1day and 7day lag for the different deep learning models

Figures 13, 14, 15 shows the MAE, RMSE, and $R^{2}$ in 1 and 7-days lags of the seven models in 32 Batch size. These values are between the predicted and true values of $PM_{2.5}$ concentration.

**Fig. 16:**
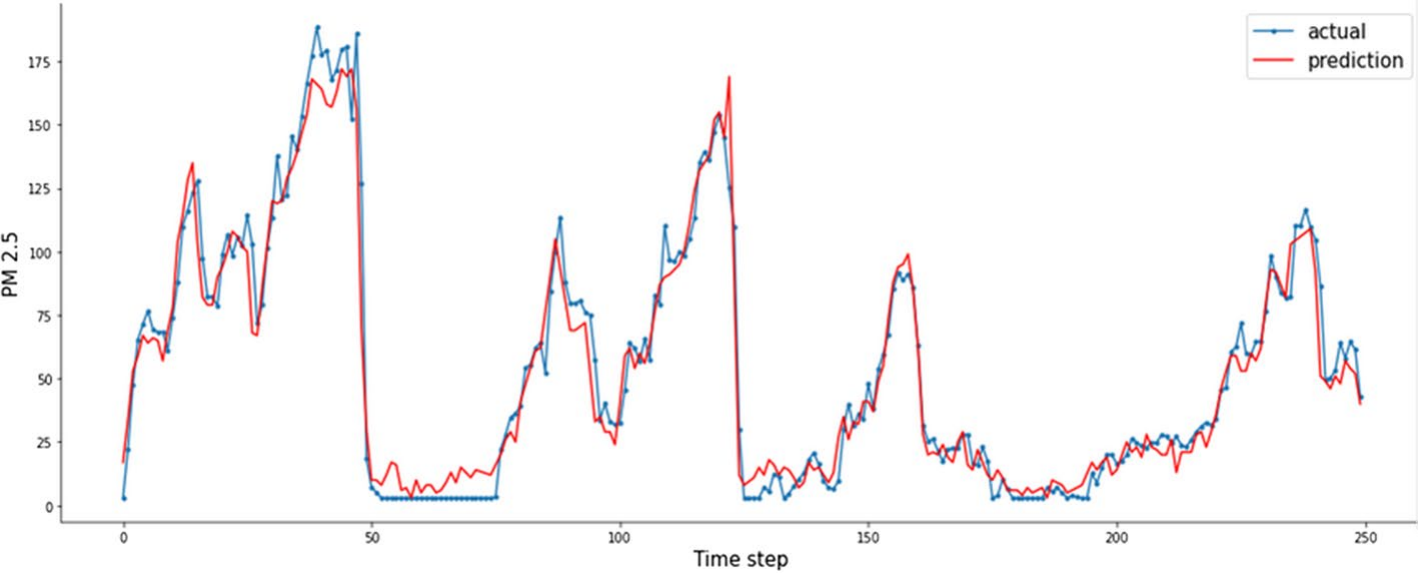
PM2.5 concentration forecasting results for 10 days

This Figure indicates that :

- By comparing CNN with LSTM in 1-day lags, the MAE and RMSE of LSTM decrease, $R^{2}$ increases, MAE decreases from 9.591 to 9.503, and RMSE decreases from 16.981 to 16.217, so LSTM was better than CNN. However, the error measurement indexes MAE and RMSE of CNN-LSTM are the smallest, and the maximum $R^{2}$ is close to 1.
- By Comparing CNN-LSTM with LSTM based on metrics MAE and RMSE. The proposed model in this paper has the smallest value of MAE and RMSE than those LSTM without the CNN layer, $R^{2}$ has a certain improvement, MAE decreases from 9.503 to 6.742, RMSE decreases from 16.217 to 12.921, and $R^{2}$ increases close to 1.
- By comparing CNN-LSTM in 1-days lags with 7-days lags, the MAE and RMSE increases, $R^{2}$ decreases, MAE increases from 6.742 to 9.034, and RMSE increases from 12.921 to 16.625 (Fig. 16).

Overall, observations from Table 1 and Figures 13, 14, and 15 show that the performance of CNN-LSTM in 1-day lags is the best among the Seven models. In terms of forecasting accuracy, MAE is 6.742 and RMSE is 12.921, which is the smallest among the seven forecasting models and has high forecasting accuracy, in terms of forecasting performance, and the $R^{2}$ of CNN-LSTM is 0.989, Therefore, the CNN-LSTM proposed in this paper is superior to the other comparative models, so the predicted value has a good explanation for the true value.

### Comparison with recent work

Four recently published models are suggested, such as AC-LSTM by [53], LSTM-FC by [33], XGBoost by [54], and CNN-LSTM by [55], which are evaluated for comparing the performance of the proposed model. Those four models were also used to forecast pollutant particles $PM_{2.5}$. The comparison investigation was using the same two metrics, MAE, RMSE.

**Fig. 17:**
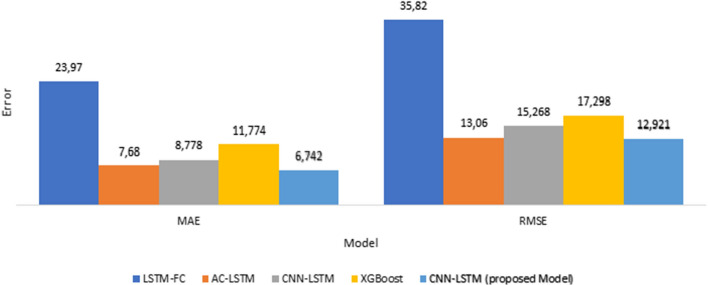
Comparison of the RMSE and MAE for the proposed model and other model

A comparative examination of MAE and RMSE, as shown in Fig. 17, shows that not only the lowest mean absolute error but also the lowest root mean square error occurs in the suggested model.

In this study, we developed a CNN-LSTM, which can effectively perform Spatio-temporal prediction, and used it to predict air quality in Beijing. The data of $PM_{2.5}$ concentration, concentrations of air pollutants highly correlated with $PM_{2.5}$, meteorological data, and $PM_{2.5}$ concentrations were collected from several locations of adjacent monitoring stations. The $PM_{2.5}$ prediction model showed high predictive accuracy and explanatory power, as well as the potential for future improvement by introducing a long-term prediction model.

- First, the CNN-LSTM prediction model can be expected to produce high $PM_{2.5}$ prediction accuracy by learning Spatio-temporal information from big data. In the case of previous prediction models, it is difficult to effectively learn Spatio-temporal information. The CNN-LSTM prediction model directly manages space-time information from adjacent stations.
- Second, we can learn effectively with the CNN-LSTM model by using data from adjacent monitoring stations. Existing air quality monitoring models have shown limitations in measuring and predicting particulate matter, due to ignorance of the effects of pollution in places not covered by the monitoring station. However, the prediction model proposed in this article can support the effects of uncovered areas.
- Third, our model was only applied in the city of Beijing in China due to the limitation of hourly open access data. In the future, the proposed model can be comprehensively evaluated by applying it to other study areas or to other time periods once the greatest amount of data is available.

However, our study has a limit. The concentrations of pollutants of foreign origin affecting Beijing were not taken into account in this study. For example, the air pollution caused by other Chinese cities is carried by the wind.

## Conclusion

In this paper, we proposed a hybrid model based on CNN and LSTM, which was used to predict the $PM_{2.5}$ of air pollutants in the urban area of Beijing. First of all, the historical data of the stations were analyzed for correlation. After experimental comparison, a feature with a higher correlation coefficient with the $PM_{2.5}$ was selected, weather data, and correlation between other stations. Secondly, based on the proposed hybrid model, we also used CNN to effectively extract the spatial characteristics of and the internal characteristics between different attributes; simultaneously, LSTM was used to obtain the time features and obtain a more accurate and stable prediction effect. Through performance evaluation and comparison of results, the main findings of this paper are as follows: this model can effectively extract the temporal and spatial features of the data through CNN and LSTM, and it also has high accuracy and stability. Due to the periodicity of the air quality data, a 24h was chosen for the input values.

## Data Availability

Not applicable. For any collaboration, please contact the authors.
